# How complex are intracellular immune receptor signaling complexes?

**DOI:** 10.3389/fpls.2012.00237

**Published:** 2012-10-23

**Authors:** Vera Bonardi, Jeffery L. Dangl

**Affiliations:** ^1^Department of Biology, University of North CarolinaChapel Hill, NC, USA; ^2^Howard Hughes Medical Institute, University of North CarolinaChapel Hill, NC, USA; ^3^Curriculum in Genetics and Molecular Biology, University of North CarolinaChapel Hill, NC, USA; ^4^Department of Microbiology and Immunology, University of North CarolinaChapel Hill, NC, USA; ^5^Carolina Center for Genome Sciences, University of North CarolinaChapel Hill, NC, USA

**Keywords:** NLR, immune system, protein complex, disease resistance, effector, plant

## Abstract

Nucleotide binding leucine-rich repeat proteins (NLRs) are the major class of intracellular immune receptors in plants. NLRs typically function to specifically recognize pathogen effectors and to initiate and control defense responses that severely limit pathogen growth in plants (termed effector-triggered immunity, or ETI). Despite numerous reports supporting a central role in innate immunity, the molecular mechanisms driving NLR activation and downstream signaling remain largely elusive. Recent reports shed light on the pre- and post-activation dynamics of a few NLR-containing protein complexes. Recent technological advances in the use of proteomics may enable high-resolution definition of immune protein complexes and possible activation-relevant post-translational modifications of the components in these complexes. In this review, we focus on research aimed at characterizing pre- and post-activation NLR protein complexes and the molecular events that follow activation. We discuss the use of new or improved technologies as tools to unveil the molecular mechanisms that define NLR-mediated pathogen recognition.

## INTRODUCTION

Plants can perceive microbial invaders through two major classes of immune receptors: surface/extracellular receptors, or intracellular immune receptors. Surface receptors, which include receptor-like kinases (RLK) and receptor-like proteins (RLP), detect both microbial-associated molecular patterns (MAMPs), typically conserved within a class of microbe, as well as specific virulence products, or effectors (Monaghan and Zipfel, 2012). Intracellular immune receptors of the nucleotide-binding domain leucine-rich repeat (NLR) protein superfamily play a central role in pathogen recognition and subsequent modulation of immune signaling in both plants and animals. The commonality of domains used by these innate immune receptors is likely the product of convergent evolution (Ausubel, 2005). Thus, NLRs across kingdoms share a common architecture that appears to reflect a common activation mechanism and, to a certain extent, common immune system output functions. Plant NLRs are critical sensors of intracellular pathogen virulence factors, or effectors, whereas their animal counterparts typically sense microbial and endogenous danger signals and link this to the activation of caspase-1 through inflammasome formation (Jones and Dangl, 2006; Franchi et al., 2012). Recent evidence from natural NLR variants and induced mutations (Hayashi et al., 2010; Kofoed and Vance, 2011; Bonardi et al., 2012) demonstrates that this set of sensor functions can be expanded to include a role for some NLRs as “helpers” that transduce signals downstream of some pathogen-activated “sensor” NLRs. As we discuss below, there may be mechanistic divergence between these two broad utilities of the NLR structural platform (Bonardi et al., 2011; Kofoed and Vance, 2011).

**FIGURE 1:**
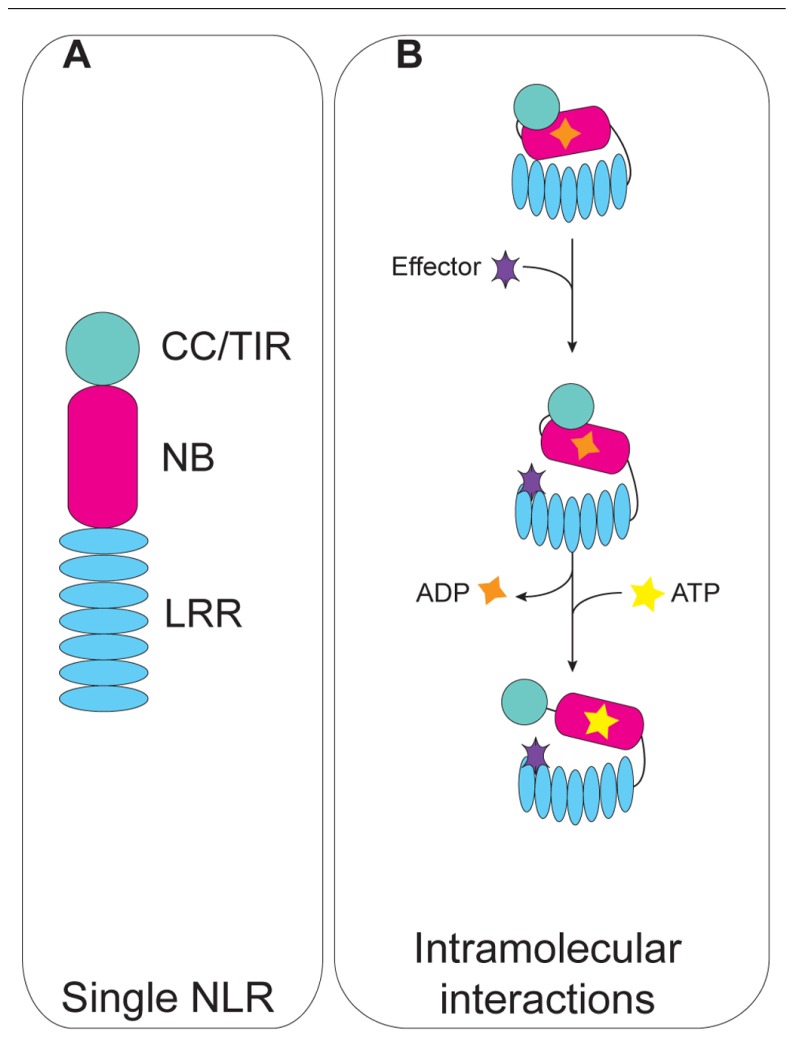
**Schematic representation of intramolecular interactions of plant NLRs**. **(A)** Domain modularity of plant NLRs. **(B)** Intramolecular interactions maintain the NLR in an “off” state through the inhibitory function of the LRR domain (top). Effector recognition results in a conformational change that allows nucleotide cycling and NLR activation (middle). Catalytic activity of the NB domain triggers a second conformational change that exposes the N-terminal domain (bottom).

NLRs consist of a central nucleotide-binding (NB) domain that modulates sensor NLR activation state through the essential catalytic P-loop motif (Takken and Tameling, 2009), and a C-terminal leucine-rich repeat (LRR) domain which is highly polymorphic and variable in the number of the repeats, and typically confers recognition specificity (**Figure 1A**). Despite a similar domain organization, NLRs are diverse in their N-termini. N-terminal variability of plant NLRs is generally limited to either a coiled-coil (CC) domain, or a Toll/interleukin-1 receptor domain (TIR); occasionally unique extended N-termini can be found in CC–NLRs and TIR–NLRs, as in the case of the tomato protein Prf (Meyers et al., 2003; Mucyn et al., 2006). Conversely, a wider range of domains at the N-termini is observed in animal NLRs (Bonardi et al., 2012).

Although NLRs were originally discovered in plants almost 20 years ago (Bent et al., 1994; Mindrinos et al., 1994), and described in animals soon thereafter (Inohara et al., 1999), the molecular mechanisms by which they sense microbial infection and subsequently transduce defense signaling remain largely elusive. Furthermore, few generalizable analogies exist among the modes of NLR regulation (Eitas and Dangl, 2010; Bonardi et al., 2012). Plant NLRs sense infection by direct recognition of the microbial effector or by sensing microbe-induced modifications of host NLR-associated proteins (Jones and Dangl, 2006). However, the microbial trigger responsible for immune signaling initiation and the molecular mechanisms that control the NLR-dependent signaling events following activation remains unknown for most NLRs.

**FIGURE 2:**
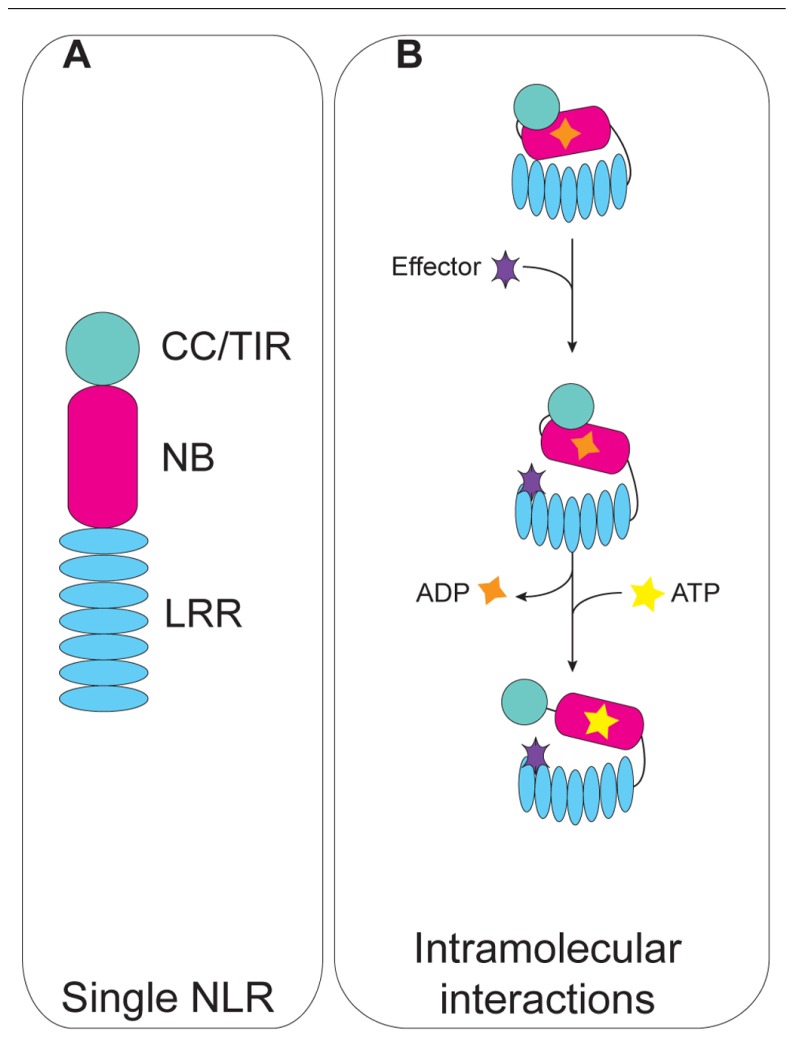
**Pre- and post-activation status of NLR immune complexes in plants**. The order of the NLRs described reflects the presentation in the text. N exists as monomers prior to activation. p50 sequesters the chloroplastic protein NRIP1 and allows association of NRIP1 to the TIR domain of N, and dimerization of N. RPS5 dimerizes in its resting state and is associated with PBS1 through the RPS5 CC domain. AvrPphB targets and cleaves PBS1, activating RPS5. MLA10 exists in inactive homodimers and recognition of the specific pathogen effector triggers nucleotide-binding/hydrolysis/exchange-dependent conformational changes that allow the recruitment of WRKY transcription factors. L6 is in an inactive monomeric state and upon AvrL567 recognition through the LRR domain, L6 self-associates into dimers through TIR–TIR domain interactions. RPS2 associates with RIN4 prior to activation; no evidence for RPS2 homodimerization exists. AvrRpt2 targets and cleaves and this relieves RIN4-dependent suppression of RPS2 activity. Resting state RPM1 is in a heteromeric protein complex that comprises the guardee RIN4. Moreover RIN4 also associates with RIPK, but whether resting state RIN4, RIPK, and RPM1 are part of the same protein complex, or not, remains unknown. AvrB or AvrRpm1 enhance RIPK-mediated phosphorylation of RIN4, and this drives nucleotide-binding/hydrolysis/exchange-dependent activation of RPM1. Prf forms homodimers that bridge Pto to Fen, or possibly another Pto-family kinase. AvrPto targets Pto and recognition results in a conformational change that activates Prf signaling. AvrPtoB is an E3 ubiquitin ligase that initiates the degradation of Fen, moreover AvrPtoB recognition by Pto results in the phosphorylation of the E3 ligase domain of AvrPtoB by Pto, thus Pto is resistant to AvrPtoB-mediated degradation. No evidence for RPS4 self-association exists, thus the RPS4 inactive state is thought to contain monomeric RPS4, EDS1, and SRFR1. Cleavage of AvrRps4 releases the C-terminus AvrRps4^C^ that interacts with EDS1, thus altering the endomembrane-associated receptor complex. Post-delivery effector processing is a common event, however it is not detailed in this review. Release of the EDS1-containing RPS4 complexes to the cytoplasm and to the nucleus is thought to activate two different defense branches: cell death, and bacterial growth-restriction respectively, and these may occur in different cellular compartments.

Here we focus on the molecular dynamics that accompany NLR activation and signaling in animals and plants. We present our review in a stepwise manner: from conserved intramolecular interactions aimed to control pre-activation NLR activity, to diverse types of post-activation multimer formation that in some cases ensures appropriate downstream signaling. We focus on molecular changes of intramolecular interactions, homotypic interactions, multimers and higher-order complexes associated to pre- and post-activation states (summarized in **Figure 2**).

## INTRAMOLECULAR INTERACTIONS

NLRs must be under tight control to prevent unnecessary ectopic activation of immune responses, which can be detrimental for growth and development. Several examples over the years support a model in which NLR activity is held in check by intramolecular interactions (Takken et al., 2006; Marquenet and Richet, 2007).

Pepper Bs2 and potato Rx were the first NLRs for which intramolecular domain–domain association was demonstrated (Moffett et al., 2002; Leister et al., 2005; Rairdan et al., 2008). While only one intramolecular association between the N-terminal (NX) and the corresponding NB domain was identified in Bs2 (Leister et al., 2005), at least two distinct interactions involving either the CC or the LRR domains with the NB domain were demonstrated for Rx (Moffett et al., 2002). It is noteworthy that intramolecular interactions of Rx are disrupted by the presence of the cognate elicitor, CP (PVX coat protein). By contrast, intramolecular interactions between the NX-NB and the LRR domains of Bs2 are not altered by recognition of the AvrBs2 effector (Leister et al., 2005). Moreover, while the Rx CC–NB interaction requires a functional P-loop (Moffett et al., 2002), the NB–LRR interaction does not. Consistent with this, tomato Mi-1.2 is capable of intramolecular interactions between the CC–NB and the LRR domains, and this association is independent of nucleotide binding activity (van Ooijen et al., 2008). Together, these indicate first, that some or all of nucleotide binding, hydrolysis and exchange are required for maintaining proper pre-activation CC–NB interactions, and second, that CP-dependent activation of Rx requires two consequent molecular rearrangements separated by a nucleotide-binding/hydrolysis/exchange event.

Like Rx, Arabidopsis RPS5 activity is also regulated by intramolecular interactions (CC–NB, NB–LRR), consistent with the hypothesis that the LRR domain maintains the protein in an inactive state to prevent ectopic NLR signaling. This resting state allows subsequent specificity for pathogen recognition (Ade et al., 2007; Bai et al., 2012; Qi et al., 2012). No direct evidence defines intramolecular interactions for barley MLA10. However, molecular dynamics simulation of its CC structure (discussed below) suggests that the EDVID motif within the CC domain (which ensures the Rx CC–NB interaction) might modulate both intra- and intermolecular interactions in MLA10 (Rairdan et al., 2008; Maekawa et al., 2011).

Pre-activation intramolecular domain–domain interactions seem to be a conserved characteristic of NLRs across kingdoms. The inactive resting state of the animal NLR-related protein Apaf-1 is achieved by stacking of the N-terminal caspase recruiting domains (CARD) against a network of intramolecular interactions within the NB domain (Riedl et al., 2005). As demonstrated by structural studies, this packed conformation limits access to the bound ADP molecule, slowing nucleotide hydrolysis/exchange. Furthermore, biophysical characterization of the mammalian NLR NOD2 revealed that the two N-terminal CARDs interact with one another, likely to cooperatively create a binding surface for partner proteins, or alternatively to maintain the NLR in an inactive state (Fridh and Rittinger, 2012).

Based on these studies, the current model for NLR activation involves three steps (**Figure 1B**; Takken et al., 2006): (i) the microbial molecule or effector, or modified-self generated by effector action on a cellular target, are respectively recognized directly or indirectly by the NLR protein; recognition triggers a first molecular rearrangement that releases the inhibitory function of the LRR domain from the NB domain; (ii) the NB becomes accessible for nucleotide exchange, and NLR activation ensues; (iii) continued nucleotide cycling drives a second conformational change that releases the N-terminal domain from the NB domain, and likely makes it accessible as a platform for interactions with downstream signaling partners.

## Homotypic Interactions

An additional feature of NLR proteins conserved among animals and plants is the potential for self-association. Increasing evidence suggests that NLR homodimers are the molecular foundation for pre-activation resting state and, in some case, for post-activation signaling events (**Figure 2**).

Potential for dimerization has been described for several plant NLRs, mainly through co-immunoprecipitation analysis of differentially epitope-tagged proteins. Although there is no evidence for self-association of the TIR–NLR N protein in its resting state, detection of the TMV elicitor p50 triggers post-activation N dimerization through association of the TIR domains (Mestre and Baulcombe, 2006; **Figure 2**). Self-association is an early post-activation event that follows pathogen recognition, as an intact P-loop is required for N dimerization (Mestre and Baulcombe, 2006). Similarly, Arabidopsis RPS5 is capable of homotypic association and each domain can interact with itself in the resting state; this association is not affected by activation (Ade et al., 2007; **Figure 2**). In this context, both N and RPS5 are thought to maintain the inactive state through intramolecular interactions, as described above. This state is perturbed by recognition of the specific elicitor, and by subsequent nucleotide cycling, which leads to exposure of the N-terminal TIR- and CC-domains, respectively. Given their diverse N terminal domains, this final event is thought to have different consequences: the N TIR domain becomes a homodimeric signaling platform; whereas the pre-existing RPS5 homodimer, newly exposed CC domains (Ade et al., 2007), might offer a binding-site for as yet unknown interacting proteins.

Given that this limited and conflicting data relies exclusively on co-immunoprecipitation assays, generalizations for the role and the dynamics of self-association may not exist. A major obstacle in the characterization of immune complexes has been the lack of reliable and robust systems to analyze NLR assembly at the molecular level. Recent technical advances begin to overcome this problem. Although to date no full-length NLR structure has been solved, the N-termini of flax L6 and barley MLA10 were recently crystallized (Bernoux et al., 2011; Maekawa et al., 2011). Both the L6 TIR domain and the MLA10 CC domain formed dimers in solution, and in both cases mutations at the dimer interface disrupted homodimerization and signaling activity. Interestingly, missense mutations in the αC helix or the BB loop in the L6 TIR prevented signaling but not dimerization, indicating the potential involvement of the relevant wild-type residues in recruiting post-activation, post-dimer formation signaling partners (Bernoux et al., 2011). Signaling by the MLA10 CC dimer is thought to mimic the microbial elicitor-activated state of the MLA10 homodimer that normally is formed following nucleotide binding/hydrolysis (Bai et al., 2012) potentially via recruitment of WRKY transcription factors for downstream signaling (Shen et al., 2007). L6 is likely to function similarly to N, since self-association does not occur in the resting state, but does accompany activation. Conversely MLA10 dimerization is effector-independent and observed in the resting state, analogous to RPS5 (**Figure 2**). This observation might indicate a functional difference between CC- (MLA10 and RPS5) and TIR- (L6 and N) NLRs. TIR–NLR resting states might be monomeric and, upon pathogen recognition, self-association might provide the N-terminal TIR dimer activation module. Conversely CC–NLRs might constitutively exist as homodimers that undergo activation-dependent conformational rearrangements to expose normally buried surfaces to anchor signaling partners. In support of this theory, the resting state CC–NLR MLA1 was also found in a high-molecular weight complex, and full-length MLA1 co-immunoprecipitated with itself (Maekawa et al., 2011). However, the CC–NLR MLA27 eluted as a monomer *in vitro* after expression and purification from insect cells (Maekawa et al., 2011), suggesting that if this hypothesis is true, homodimerization *in vivo* is mediated by plant-encoded assembly machinery. In light of this evidence, it is tempting to speculate that homodimerization might represent an essential molecular mechanism for the downstream signaling rather than for effector recognition, as indicated by the fact that TIR–NLR homodimerization typically follows NLR activation.

Tomato Prf is a CC–NLR with a unique N-terminal domain that conditions recognition of the bacterial effectors AvrPto and AvrPtoB via its interaction with the host Ser/Thr kinase Pto. While the Pto homolog Fen is marked for degradation by AvrPtoB through the activity of the effector’s E3 ubiquitin ligase domain (Rosebrock et al., 2007), Pto itself is resistant to AvrPtoB-dependent degradation. This is because Pto phosphorylates and thus inactivates the AvrPtoB E3 ligase domain (Ntoukakis et al., 2009). Independently, structure-based functional analysis suggested that binding of AvrPto to Pto alters the conformation of Pto, thereby releasing inhibition of Prf and allowing its NB-dependent activation (Xing et al., 2007). Prf is capable of homodimerization, as shown by co-immunoprecipitation; this interaction is primarily mediated by the Prf N-terminal domain and is independent of Pto and AvrPto (Gutierrez et al., 2010), indicating that self-association, similarly to MLA, is constitutive and occurs prior to pathogen detection (**Figure 2**).

Self-association of mammalian NLRs has been widely demonstrated and is a common post-activation event (Hu et al., 1998; Kobayashi et al., 2002; Inohara and Nunez, 2003). Here, NLR activation typically results in inflammasome formation. This physical interaction aids the recruitment of pro-caspase-1 through its direct CARD–CARD interaction with the NLR protein or, in the case of PYD–NLR, through the adaptor protein ASC which bridges the pro-caspase-1 to the inflammasome NLR (Rathinam et al., 2012). Interestingly, homotypic interactions of mammalian NLRs are mediated by the NACHT/NB domain, whereas the diverse N-terminal domains seem to mediate interaction with accessory proteins for the downstream immune signaling (Rathinam et al., 2012). This highlights a potential difference in comparison to the self-association mechanisms adopted by plant immune NLR receptors noted above.

## HETEROTYPIC INTERACTIONS

Although structural and functional similarities exist among NLRs within and across kingdoms, mechanistic regulation might rest only on intramolecular interactions to regulate activation, and homotypic interactions to modulate subsequent signaling.

Protein–protein interactions are the foundation of pathogen detection at least for plant sensor NLRs, as initiation of immune responses typically follows direct or indirect association between the NLR and the microbial product. Arabidopsis RPP1 and flax L6 specifically recognize the oomycete ATR1 and the fungal AvrL567 effector, respectively. In both cases, direct interactions are thought to be determined by the LRR domain as suggested by co-immunoprecipitations of the RPP1 LRR domain to ATR1, or by interaction in yeast as well as structural and mutational analysis for L6 (Dodds et al., 2006; Wang et al., 2007; Krasileva et al., 2010). Direct interaction between pathogen effector and the corresponding immune receptor was recently described in rice, where the *Magnaporthe oryzae* AVR-Pik was found to physically bind the CC domain of Pik (Kanzaki et al., 2012). Plant NLRs can also be activated by effector-induced modifications of an associated host target, as suggested by the guard hypothesis (Van der Biezen and Jones, 1998; Dangl and Jones, 2001). The Arabidopsis CC–NLR RPS5 determines recognition of the bacterial effector AvrPphB, but this event is mediated by the host protein kinase PBS1 (Shao et al., 2003). PBS1–RPS5 physical interaction is a pre-activation event and PBS1 cleavage by the cysteine protease effector AvrPphB is required for RPS5 activation. This presumably causes conformational rearrangements that allow nucleotide exchange (Ade et al., 2007; DeYoung et al., 2012; Qi et al., 2012; **Figure 2**).

Similarly, the Arabidopsis CC–NLRs RPS2 and RPM1 physically associate with different cellular pools of membrane associated RIN4, which is differentially targeted by multiple effectors. AvrB-mediated phosphorylation of RIN4, likely by the receptor-like kinase RIPK (Chung et al., 2011; Liu et al., 2011), or cleavage by the cysteine protease effector AvrRpt2 (Kim et al., 2005) is necessary and sufficient to convert resting state RPM1 and RPS2 respectively into signaling active states (**Figure 2**). Thus, PBS1 and RIN4 function as guardees for the corresponding immune receptors, RPS5 and either RPM1 or RPS2.

Tobacco NRIP1 is a chloroplastic protein that mediates the indirect association of p50 to the TIR domain of the N immune receptor (Caplan et al., 2008). However, NRIP1-N association is not constitutive, but rather requires the formation of the pre-recognition complex of NRIP1 (**Figure 2**). Hence NRIP1–N his interaction might reflect a novel mechanism required for activation of defense signaling where the immune receptor monitors an effector-dependent relocalization of a pre-recognition host complex as a modified self.

Recent high-throughput studies unveiled the potential for protein–protein interactions between immune components. Both an *Arabidopsis* interactome network and the plant-pathogen protein–protein network were identified through yeast-two-hybrid (Arabidopsis Interactome Mapping Consortium, 2011; Mukhtar et al., 2011). Although the obvious constraints of this heterologous system apply, the network revealed a greater than random propensity for indirect interaction between pathogen effectors and host immune receptors. However, whether these indirect interactions reflect a genuine gene-for-gene interaction, or whether they may be biased toward the identification of effector targets over the effector-specific immune receptor remains elusive in the absence of further validation.

## HIGHER-ORDER COMPLEXES

Higher-order complex formation has been demonstrated for some plant NLRs, as we discuss below, and is well established for animal NLRs (Franchi et al., 2012). However requirements for diverse accessory partners, and variable oligomer stoichiometry are, thus far, the norm, suggesting the existence of diverse NLR immune complexes that could reflect different activation and/or signaling mechanisms (**Figure 2**).

As described above, tomato Prf is part of a high-molecular complex that contains a Prf dimer, which can bridge Pto and Fen (Gutierrez et al., 2010). This complex likely functions as a regulatory switch to control immune responses and is activated via effector-dependent disruption of negative regulation on Prf (Mucyn et al., 2006, 2009; Gutierrez et al., 2010). Size-exclusion gel filtration analysis combined with mass spectrometry (MS) on the immunoaffinity purified Prf complex allowed the identification of a hetero-multimer that is likely to contain two Prf molecules and two Pto-family kinases (Gutierrez et al., 2010). Additional data suggest that Prf is capable of homotypic and heterotypic interactions with at least Pto and Fen, although two additional Pto-family kinases, Pth2 and Pth3, were also found to be associated with the Prf complex as well (Gutierrez et al., 2010). This multimerization event is thought to bring into close proximity the Prf-associated kinases and thus likely can broaden the specificity of effector recognition events that can activate Prf.

Notably, Prf-dependent defense signaling reflects a pathogen detection mechanism that does not conform simply to the guard hypothesis. Although AvrPto and AvrPtoB recognition leads to Prf-dependent effector-triggered immunity (ETI), these effectors also physically interact with pattern recognition LRR-kinase receptors (PRRs) that typically regulate MAMP-triggered immunity (MTI; Ausubel, 2005), hence, Pto-family kinases and PRR kinases are co-receptors for common effectors. In this context, Prf-mediated ETI evolved as a mechanism to intercept and re-direct effector-triggered suppression of MTI responses into effective ETI (Gohre et al., 2008; Xiang et al., 2008; Gimenez-Ibanez et al., 2009).

Increasing evidence suggests that both products of head-to-head NLR gene pairs are often required for full disease resistance (Eitas and Dangl, 2010; Okuyama et al., 2011). One of the best characterized and most appealing of such cases is the *Arabidopsis* dual resistance gene system of RRS1–RPS4. RRS1-R is an atypical NLR that contains a C-terminal WRKY domain and confers resistance to *Ralstonia solanacearum* expressing PopP2 effector. RPS4 is a TIR–NLR. Physical association between RRS1 and PopP2 has been demonstrated both in yeast cells and in the nucleus of living plant cells (Deslandes et al., 2003; Tasset et al., 2010). Intriguingly, both RRS1 and RPS4 are required for specific AvrRps4-triggered immunity, as well as resistance to *R. solanacearum* and *Colletotrichum higginsianum* (Birker et al., 2009; Narusaka et al., 2009). RPS4 is capable of shuttling to the nucleus and RRS-R levels are enhanced in the nucleus in the presence of PopP2 (Deslandes et al., 2003; Wirthmueller et al., 2007). Thus, an attractive possibility is that effector-mediated activation could result in a yet to be detected physical interaction between RRS1 and RPS4 in the nucleus. This interaction would, in turn, promote transcriptional regulation of target genes.

Although no current evidence for Arabidopsis RPS4 homodimerization exists, it is tempting to speculate that the RPS4–RRS1 heterodimer could be the functional molecule (see above). In addition, the RPS4 TIR–NLR was recently found to physically associate with an immune regulator of basal defense and ETI, ENHANCED DISEASE SUSCEPTIBILITY1 (EDS1; (Bhattacharjee et al., 2011; Heidrich et al., 2011). In this context EDS1 is potentially an adaptor protein that could connect effector recognition to downstream defense pathways. Interestingly EDS1 is capable of physical interaction not only with RPS4, but also with the unrelated TIR–NLR RPS6, and the likely transcriptional repressor, SRFR1. However, recognition of the RPS4 and RPS6 activating effectors, AvrRps4 and HopA1 respectively, apparently disrupts the EDS1 interaction with SRFR1 and the TIR–NLR receptors (Bhattacharjee et al., 2011; **Figure 2**). These authors suggested that the association of an NLR immune receptor with EDS1 might underpin a novel mechanism for immune responses where a basal defense regulator coordinates various immune responses that are both effector-dependent and -independent. Thus, EDS1 could be a common virulence target guarded by a number of TIR–NLRs. However, Sohn et al. (2012) were recently unable to reproduce co-immunoprecipitation between AvrRps4 and EDS1. Thus, it remains uncertain whether EDS1 is a *bona fide* guardee.

Besides the large number of heterotypic interactions described for animal NLRs with accessory proteins involved in inflammasome formation, new evidence suggests that NLRs themselves can, in some cases, heterodimerize. The NLRC4 inflammasome is activated by bacterial flagellin and type III secretion system component PrgJ. Recognition of flagellin is specifically mediated by the sensor NLR NAIP5, whereas the NLR NAIP2 serves as the sensor NLR for PrgJ (Kofoed and Vance, 2011; Zhao et al., 2011). The NAIP NLR sensors control ligand-dependent NLRC4 oligomerization in a similar manner: flagellin recognition results in NAIP5–NLRC4 heteromerization, whereas PrgJ recognition drives NAIP2–NLRC4 association (Kofoed and Vance, 2011; Zhao et al., 2011). Thus, NLRC4 is a “helper” NLR for the function of at least two sensor NLRs (Rathinam et al., 2012).

Plant NLR functions were also recently differentiated into “helper” or “sensor” (Bonardi et al., 2011, 2012). Tomato NRC1 and tobacco NRG1 represent the first examples of NLR proteins that function as helper for either Cf-4 or N respectively (Peart et al., 2005; Gabriels et al., 2007). The Arabidopsis CC–NLR ADR1-L2 can act as a helper NLR that regulates signal transduction following effector detection via at least two sensor NLRs. Intriguingly neither ADR1-L2 nor NLRC4 require a functional nucleotide-binding domain to fulfill their helper NLR functions. This suggests that helper NLRs might share common signaling mechanisms (Bonardi et al., 2011; Kofoed and Vance, 2011). A non-functional P-loop variant of NLRC4 abolished both homodimerization and heteromerization with NAIP5 (Zhao et al., 2011), but retained inflammasome-dependent cell death signaling (Kofoed and Vance, 2011). This result could indicate that the non-functional P-loop NLRC4 mutant is unable to coordinate nucleotide binding, but might still retain the ability to function as an adaptor to recruit CASP1 and activate immune signaling downstream from the activated sensor NLRs. Similar to NLRC4, ADR1-L2 might function as an adaptor for effector-activated sensor NLRs, although no clear mechanism exists yet. Because ADR1-L2 coordinates several sorts of immune responses, from effector-dependent to the recognition of conserved microbial compounds, we speculate that various triggers of the plant defense output response might converge on ADR1-L2, possibly via direct physical interaction of this NLR with other defense machinery components. Moreover, the rice Pb1 NLR family, which naturally lacks a P-loop motif, conditions broad spectrum resistance to rice blast, potentially by acting as a helper NLR (Hayashi et al., 2010). There are Pb1 homologs in maize, suggesting evolutionary conservation of function. Further, *Arabidopsis* and *A. lyrata* express NLR proteins that carry degenerate P-loop mutations that are likely to impair the canonical P-loop-dependent activation mechanism (Bonardi et al., 2012). These examples do not fit the current mechanistic activation paradigm outlined above, which relies on nucleotide exchange and hydrolysis to drive intra- and intermolecular rearrangements and activation. Together, these examples support a role of helper NLRs as components of a scaffold machinery for immune responses, and provide a potential mechanistic rationale for the occurrence of co-functional head-to-head NLR genes described above.

A link between the two different receptor tiers of plant immune response signaling was also recently proposed. The immune complex associated with the low abundance plasma membrane localized CC–NLR RPS2 was immunopurified and additional components were identified through chemical cross-linking and MS (Qi and Katagiri, 2011; Qi et al., 2011a,b). Interestingly, RPS2 was found to physically associate with the flagellin receptor FLS2, a PRR that regulates MTI. Furthermore FLS2 was also shown to associate with RPS5 and RPM1 in this system, suggesting that ETI and MTI signaling might be connected (Qi et al., 2011a).

## IMPLICATIONS FOR FORMATION OF DIVERSE IMMUNE PROTEIN COMPLEXES

Despite similar autoregulatory mechanisms for pre-activation of NLRs, we hope to have highlighted how variable NLR immune complexes can be. Moreover, in certain cases an NLR might interact with a wide array of partners, although the size of the associated complex thus far observed is likely too small to explain all the possible interactions (Gutierrez et al., 2010). While some of these interactions might be promiscuous and not biologically relevant, many are likely to be associated with differential signaling dynamics.

It was recently reported that plant NLR-mediated cell death, which is a hallmark of successful ETI, and disease resistance measured by pathogen growth restriction, can be uncoupled and that this bifurcation might rest on differential compartmentalization for each signaling branch (Coll et al., 2010; Heidrich et al., 2011; Bai et al., 2012). Therefore, it is straightforward to speculate that an NLR might recruit different partners depending on its cell compartment-specific function. Thus, nucleocytoplasmic partitioning of NLR-containing complexes could result from a different network of interactions (Burch-Smith et al., 2007; Shen et al., 2007; Wirthmueller et al., 2007; Cheng et al., 2009; Slootweg et al., 2010; Tameling et al., 2010). However, in the absence of insights into cell molecular dynamics during the immune response, the relevance of each interaction cannot yet be assessed.

## PROSPECTIVE

An increasing amount of evidence suggests the existence of higher-order molecular complexes associated with NLR proteins. However, whether these interactions are biologically relevant in plant or animal innate immunity cannot be assessed given the limitations of the techniques used in the majority of these reports. Co-immunoprecipitations from complex mixtures do not discriminate indirect from direct protein–protein interactions, and do not provide evidence for stoichiometry of the molecular complex. Moreover, simple protein–protein interactions do not describe the dynamics of the signaling network upon NLR activation.

Proteomics offers a powerful and indispensable technology in biology as it aids not only in the identification of the components of protein complexes, but also in the determination of post-translational modifications that might shed light on regulatory molecular mechanisms of immune signaling. A large-scale survey of the comparative identification of phosphorylation sites was recently described for plants (Nakagami et al., 2010). Interestingly, many phosphorylated residues in conserved NLR motifs essential for their function were identified, suggesting that phosphorylation might be key for NLR activation and signaling. In the past, MS workflows have been widely improved in their utility and performance, additionally the growing MS-database acquired over the years is deposited in accessible databases (Schmidt et al., 2009; Joshi et al., 2011). Besides Prf and RPS2, as we outlined above, MS on immunoaffinity purified complexes was successfully employed for the identification of novel components of the RIN4 immune complex (Liu et al., 2009). However, one of the main limitations of MS in protein complexes identification rests on how to increase sensitivity to allow monitoring of low-abundance proteins. Recent technical advances for the isolation of low abundance plasma membrane-associated NLRs might be helpful to overcome this limitation (Qi and Katagiri, 2011; Elmore et al., 2012). Moreover, quantification of peptides based on ion abundance rather than spectra counting provides a higher dynamic range of quantification (Patel et al., 2009).

Although MS provides a valuable tool to resolve immune protein complexes, it does not allow elucidation of the molecular mechanisms of protein networks. Only by exploring the 3D structure of the individual NLR proteins, and NLR proteins in complex with effectors and partners, we will be able to investigate their molecular function, to define their direct interaction with additional signaling components, and to provide mechanisms for their control, thus linking NLR structure to a biological relevant signaling system.

As more evidence on protein–protein interactions in innate immune complexes is gathered, we need to critically evaluate not only the validity of the interaction but also the physiological significance of it. Newly emerging fluorescent protein technologies represent an appealing tool to overcome this challenge and to study the spatio-temporal dynamics of the immune receptors in living cells (Miyawaki, 2011). Single molecule fluorescence will greatly advance our understanding of how immune complexes are formed and regulated, as we will likely be able to simultaneously assess protein–protein interaction and protein movement in the cell. These state of the art technologies will direct us toward new questions that can be addressed at spatiotemporal resolution: how is an immune complex formed? How is it regulated upon microbial recognition? How is the complex distributed in the cell upon activation?

Given the likely and unanticipated diversity of NLR functions, it is now essential to elucidate the molecular dynamics of immune complex formation and signaling in a variety of contexts to unveil the spectrum of different mechanisms that regulate NLR activities. Thus resolving the complexity of the NLR immune complexes remains one of the major challenges we face in order to rationally deploy NLR proteins to combat old and emerging plant diseases.

## Conflict of Interest Statement

The authors declare that the research was conducted in the absence of any commercial or financial relationships that could be construed as a potential conflict of interest.
